# Quantitative anatomy of the growing clavicle in the human fetus: CT, digital image analysis, and statistical study

**DOI:** 10.1007/s00276-017-1821-3

**Published:** 2017-02-10

**Authors:** Marcin Wiśniewski, Mariusz Baumgart, Magdalena Grzonkowska, Bogdan Małkowski, Piotr Flisiński, Małgorzata Dombek, Michał Szpinda

**Affiliations:** 1 Department of Normal Anatomy, The Ludwik Rydygier Collegium Medicum in Bydgoszcz, 1 Łukasiewicza Street, Bydgoszcz, 85-821 Poland; 2 Department of Positron Emission Tomography and Molecular Imaging, The Ludwik Rydygier Collegium Medicum in Bydgoszcz, The Nicolaus Copernicus University in Toruń, 1 Łukasiewicza Street, 85-821 Bydgoszcz, Poland

**Keywords:** Clavicle, Size, Growth dynamics, Digital image analysis, Human fetus, Regression analysis

## Abstract

**Purposes:**

Knowledge of dimensions of fetal long bones is useful in both the assessment of fetal growth and early detection of inherited defects. Measurements of the fetal clavicle may facilitate detection of numerous defects, e.g., cleidocranial dysplasia, Holt–Oram syndrome, Goltz syndrome, and Melnick–Needles syndrome.

**Methods:**

Using the methods of CT, digital image analysis, and statistics, the size of the growing clavicle in 42 spontaneously aborted human fetuses (21 males and 21 females) at ages of 18–30 weeks was studied.

**Results:**

Without any male–female and right–left significant differences, the best fit growth models for the growing clavicle with relation to age in weeks were as follows: *y* = −54.439 + 24.673 × ln(age) ± 0.237 (*R* ^2^ = 0.86) for length, *y* = −12.042 + 4.906 × ln(age) ± 0.362 (*R* ^2^ = 0.82) for width of acromial end, *y* = −4.210 + 2.028 × ln(age) ± 0.177 (*R* ^2^ = 0.77) for width of central part, *y* = −4.687 + 2.364 × ln(age) ± 0.242 (*R* ^2^ = 0.70) for width of sternal end, *y* = −51.078 + 4.174 × ln(age) ± 6.943 (*R* ^2^ = 0.82) for cross-sectional area, and *y* = −766.948 + 281.774 × ln(age) ± 19.610 (*R* ^2^ = 0.84) for volume.

**Conclusions:**

With no sex and laterality differences, the clavicle grows logarithmically with respect to its length, width, and volume, and linearly with respect to its projection surface area. The obtained morphometric data of the growing clavicle are considered normative for their respective weeks of gestation and may be of relevance in the diagnosis of congenital defects.

## Introduction

Knowledge of dimensions of fetal long bones is useful in both the assessment of fetal growth and early detection of inherited defects. Overall, abnormalities of the fetal clavicle are rare and may exemplify cleidocranial dysplasia, Holt–Oram syndrome, Goltz syndrome, and Melnick–Needles syndrome [1, 2].

The process of clavicle ossification is the earliest among all long bones. It begins at the end of the 6th week of fetal life, which determines the early development of the upper limb, ensuring its adequate mobility [3–6]. The clavicle has two primary ossification centers that fuse in its central part on day 45 [2, 7]. The sternal end of the clavicle develops most slowly, thus undergoing ossification as the very last of all bones. Developmental anatomy and morphometric data provide clinicians of different specialties with relevant information [3]. In archeological studies, the clavicular model is germane to elucidate some mechanisms of evolution, while, in forensic medicine, it serves to determine sex, age, ethnic differences, and body posture [8, 9].

A review of literature concerning morphometric studies of the clavicle revealed that different imaging methods have consecutively been engaged, namely X-rays, ultrasound, and 3D-ultrasound. In this study, based on much more advanced and objective research methods (CT, digital image analysis), we decided to perform a comprehensive morphometric analysis of the clavicle in the human fetus.

The precise purposes of the study were to:

- carry out morphometric analysis of the fetal clavicle with respect to its linear, planar, and spatial parameters to determine their normative values
- assess possible sex differences regarding all analyzed parameters, and
- establish growth dynamics for all analyzed parameters, including mathematical best-matched models for fetal age in weeks.

## Materials and methods

The study material was 42 human fetuses (21 males and 21 females) aged 18–30 weeks, originating from spontaneous abortions and preterm deliveries. As a prerequisite, with the use of CT and morphological examinations, the sample was built by rejection of malformed fetuses with conspicuous internal or external macroscopic abnormalities, and so could be deliberated normal. The fetuses were acquired before the year 2000 and remained part of the specimen collection of the Department of Normal Anatomy of our university. The study was approved by the Bioethics Committee of the Ludwik Rydygier Collegium Medicum in Bydgoszcz (KB 275/2011). The fetal age was determined based on the crown-rump length. Table 1 lists the characteristics of the study group, including age, number, and sex of the fetuses.

**Table 1:** Age, number, and sex of the fetuses studied

Gestational age	Crown-rump length (mm)				Number of fetuses	Sex	
Weeks (Hbd-life)	Mean	SD	Min	Max		♂	♀
18	133.33	5.80	130.0	140.0	3	1	2
19	150.00	3.03	146.0	154.0	6	2	4
20	159.67	0.58	159.0	160.0	3	2	1
21	174.67	3.51	171.0	178.0	3	2	1
22	186.00		186.0	186.0	2	0	2
23	196.33	1.15	195.0	197.0	3	1	2
24	208.67	3.81	204.0	213.0	9	5	4
25	214.00		214.0	214.0	1	0	1
26	229.00	5.70	225.0	233.0	2	1	1
27	239.25	2.36	236.0	241.0	4	4	0
28	249.50	0.70	249.0	250.0	2	0	2
29	253.00		253.0	253.0	1	0	1
30	263.67	1.15	263.0	265.0	3	3	0
Total					42	21	21

**Fig. 1:**
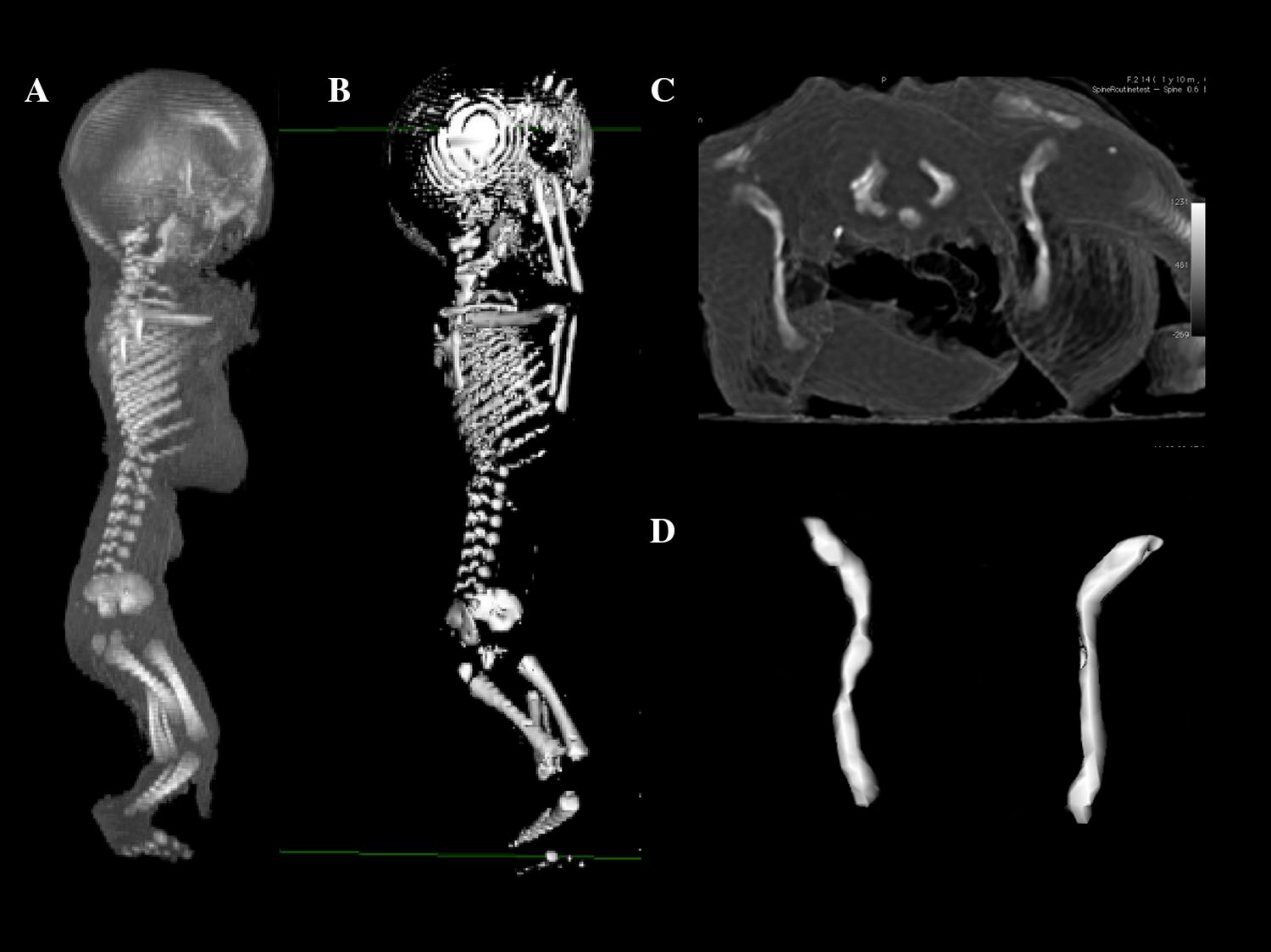
CT of a male fetus aged 24 weeks in the sagittal projection recorded in DICOM formats (**a**), 3D fetus reconstruction in the sagittal projection (**b**), its clavicle in the horizontal projection (**c**), and 3D clavicle reconstruction in the horizontal projection assessed by Osirix 3.9 (**d**)

Using the Siemens-Biograph 128 mCT camera, the fetuses were scanned at a step of 0.4 mm, recorded in DICOM formats, and subsequently subjected to morphometric analysis using the Osirix 3.9 software. Of note, Osirix 3.9 allows conducting any type of linear, planar, and three-dimensional reconstructions of the studied objects along with their precise quantitative analysis (Fig. 1).

**Fig. 2:**
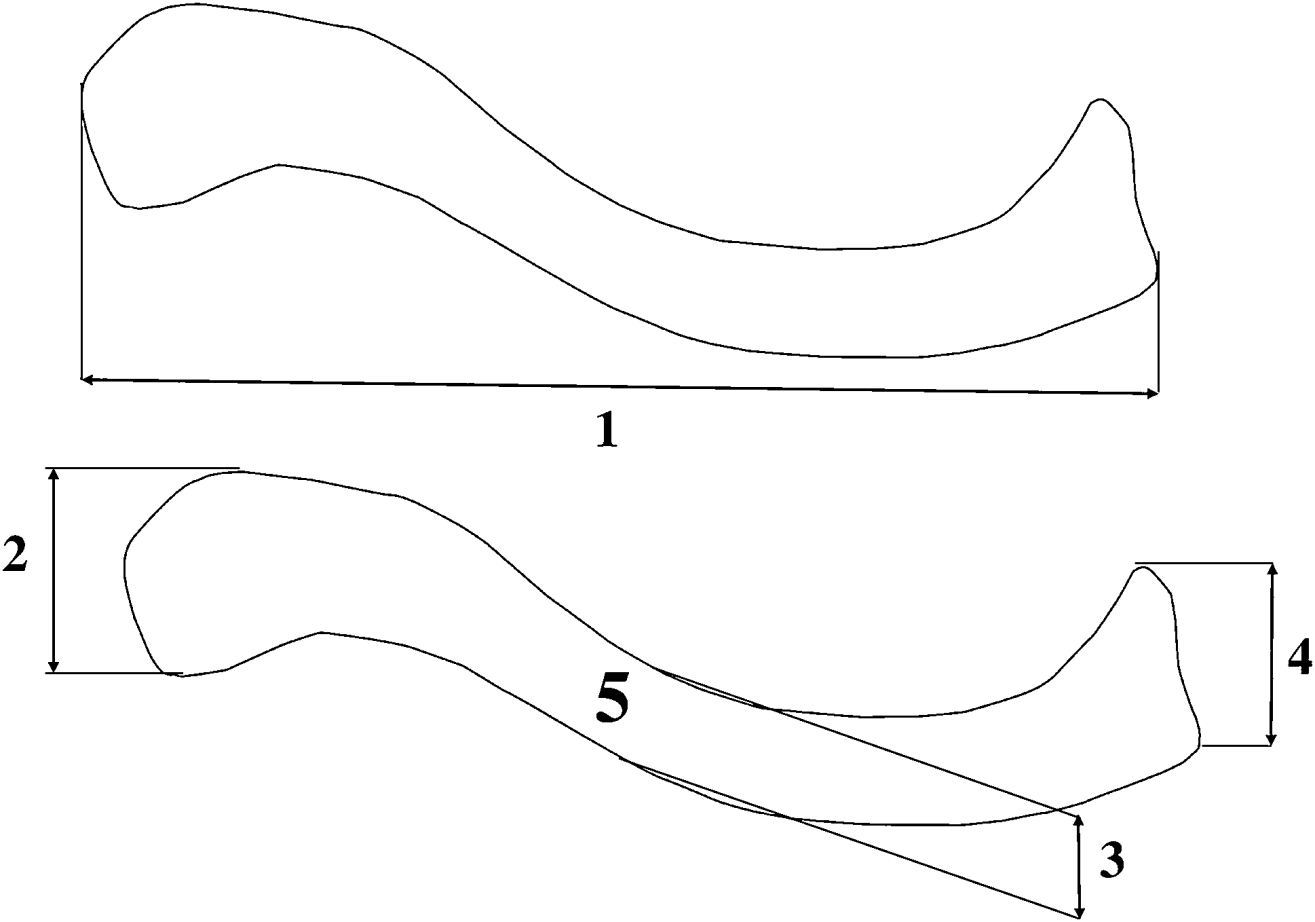
Diagram showing measurements of the clavicle in the horizontal projection: *1* length, *2* width of the acromial end, *3* width of the central part, *4* width of the sternal end, *5* projection surface area

**Fig. 3:**
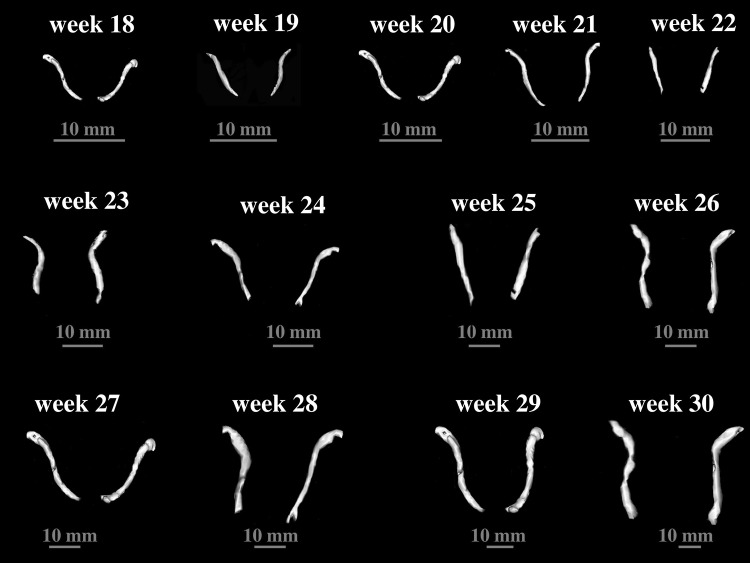
3D reconstruction of the right and left clavicles in fetuses aged 18–30 weeks assessed by Osirix 3.9

In all fetuses, quantitative assessment of the following six parameters for either clavicle (Figs. 2, 3) was performed:

1. length of the clavicle (mm), equal to the distance between its acromial and sternal ends in the transverse plane,
2. width of the clavicle acromial end (mm), equal to the distance between its anterior and posterior borderlines in the transverse plane,
3. width of the clavicle central part (mm), equal to the distance between its anterior and posterior borderlines in the transverse plane,
4. width of the clavicle sternal end (mm), equal to the distance between its anterior and posterior borderlines in the transverse plane,
5. projection surface area of the clavicle (mm^2^), based on the determined contour of the clavicle in the transverse plane,
6. volume of the clavicle (mm^3^), based on advanced spatial reconstruction of images.

In a continuous effort to minimalize measurement and observer bias, all measurements were achieved by one researcher (M.W.) and verified by the same examiner. Each measurement was done three times under the same settings but at different times, and then averaged. The intra-observer variation between repeated measurements was assessed by ANOVA and post-hoc RIR Tukey test. The numerical data were statistically analyzed. Distribution of variables was checked using the Shapiro–Wilk (W) test, while homogeneity of variance was checked using Fisher’s test. Since the fetuses studied were inadequately dispersed with fetal age, we tested sex (Student t test for unpaired variables) and laterality (Student t test for paired variables) differences for the whole sample, without considering fetal ages. To compare whether means change with age, Student’s *t* test for independent variables, and one-way analysis of variance with post-hoc Tukey’s test were used. If no similarity of variance occurred, the non-parametric Kruskal–Wallis test was used. The characterization of growth dynamics for the analyzed parameters was based on linear and curvilinear regression analysis. The match between the estimated curves and fetal age in weeks was evaluated based on the coefficient of determination (*R* ^2^).

## Results

**Fig. 4:**
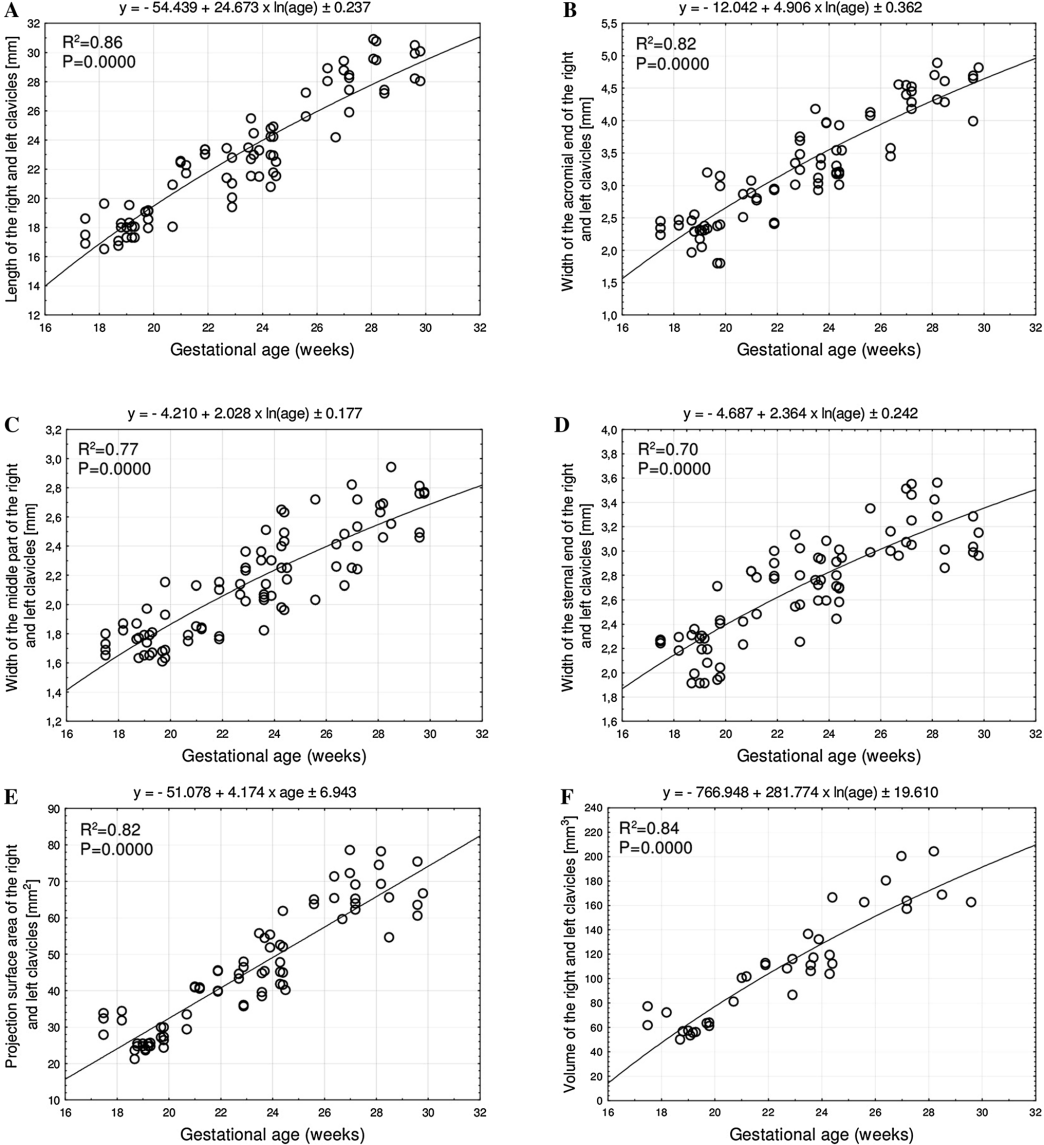
Regression lines for length (**a**), width of the acromial end (**b**), width of the central part (**c**), width of the sternal end (**d**), projection surface area (**e**), and volume (**f**) of the clavicle

No statistically significant differences (*p* > 0.05) in assessing intra-observer reproducibility of clavicle measures were found. The morphometric values obtained were characterized by normality of distribution and homogeneity of variance. As a consequence, numerical variables have been expressed as mean ± standard deviation. The lengths and three widths of the fetal clavicles have separately been presented for males (Table 2) and females (Table 3), and their projection surface areas and volumes have separately been displayed for males (Table 4) and females (Table 5). The statistical analysis for the whole sample revealed neither sex nor bilateral differences (*p* > 0.05) regarding all analyzed parameters. Therefore, we aggregately investigated the growth dynamics of the established parameters, as functions of fetal age in weeks, without considering sex or side. Thus, all individual data—not only their means—irrespective of sex and side were involved in the six regression formulae. An increase in length and width of the fetal clavicle at varying ages in weeks followed natural logarithmic functions. Between weeks 18 and 30, the mean length of the clavicle increased from 16.97 ± 0.49 to 28.70 ± 1.54 mm on the right and from 18.94 ± 0.59 to 29.39 ± 0.21 mm on the left, following the function *y* = −54.439 + 24.673 × ln(age) ± 0.237 (*R* ^2^ = 0.86)—(Fig. 4a). At the same period, the mean width of the clavicle acromial end increased from 2.34 ± 0.12 to 4.20 ± 0.54 mm on the right, and from 2.46 ± 0.09 to 4.48 ± 0.43 mm on the left, following the function: *y* = −12.042 + 4.906 × ln(age) ± 0.362 (*R* ^2^ = 0.82)—(Fig. 4b). The mean width of the clavicle central part increased from 1.77 ± 0.07 to 2.69 ± 0.17 mm on the right and from 1.75 ± 0.11 to 2.66 ± 0.17 mm on the left, following the function *y* = −4.210 + 2.028 × ln(age) ± 0.177 (R^2^ = 0.77)—(Fig. 4c). The mean width of the clavicle sternal end increased from 2.23 ± 0.04 to 3.23 ± 0.29 mm on the right and from 2.32 ± 0.06 to 3.09 ± 0.17 mm on the left, following the function *y* = −4.687 + 2.364 × ln(age) ± 0.242 (*R* ^2^ = 0.70)—(Fig. 4d).

**Table 2:** Length and widths of the fetal clavicle in males

Gestational age (weeks)	*N*	Morphometric parameters of the male clavicle															
		Length (mm)				Width of acromial end (mm)				Width of central part (mm)				Width of sternal end (mm)			
		Right		Left		Right		Left		Right		Left		Right		Left	
		Mean	SD	Mean	SD	Mean	SD	Mean	SD	Mean	SD	Mean	SD	Mean	SD	Mean	SD
18	1	16.90	–	18.59	–	2.23	–	2.44	–	1.69	–	1.73	–	2.24	–	2.27	–
19	2	17.03	0.37	17.54	0.67	2.07	0.15	2.38	0.10	1.78	0.02	1.76	0.16	1.91	0.00	2.30	0.02
20	2	18.86	0.41	18.51	0.82	2.47	0.95	2.69	0.42	1.92	0.33	1.78	0.21	2.00	0.06	2.42	0.02
21	2	22.08	0.52	22.40	0.21	2.83	0.08	2.94	0.19	1.99	0.21	1.84	0.01	2.81	0.04	2.66	0.25
22	0	–	–	–	–	–	–	–	–	–	–	–	–	–	–	–	–
23	1	23.41	–	21.36	–	3.34	–	3.01	–	2.07	–	2.14	–	3.13	–	2.54	–
24	5	23.87	1.00	22.64	1.18	3.53	0.53	3.55	0.48	2.25	0.20	2.15	0.15	2.77	0.12	2.86	0.20
25	0	–	–	–	–	–	–	–	–	–	–	–	–	–	–	–	–
26	1	25.61	–	27.21	–	4.12	–	4.07	–	2.72	–	2.03	–	3.35	–	2.99	–
27	4	26.39	2.90	27.31	2.24	4.41	0.11	4.41	0.17	2.64	0.16	2.26	0.11	3.44	0.13	3.08	0.12
28	0	–	–	–	–	–	–	–	–	–	–	–	–	–	–	–	–
29	0	–	–	–	–	–	–	–	–	–	–	–	–	–	–	–	–
30	3	28.70	1.54	29.39	1.04	4.20	0.54	4.48	0.43	2.69	0.17	2.66	0.17	3.23	0.29	3.09	0.17

Since the fetuses did not represent adequate samples for statistical analysis between particular 1-week intervals, we tested sex and laterality differences for the whole sample, without considering fetal ages

**Table 3:** Length and widths of the fetal clavicle in females

Gestational age (weeks)	*N*	Morphometric parameters of the female clavicle															
		Length (mm)				Width of acromial end (mm)				Width of central part (mm)				Width of sternal end (mm)			
		Right		Left		Right		Left		Right		Left		Right		Left	
		Mean	SD	Mean	SD	Mean	SD	Mean	SD	Mean	SD	Mean	SD	Mean	SD	Mean	SD
18	2	17.01	0.69	19.11	0.72	2.40	0.08	2.47	0.13	1.81	0.01	1.76	0.16	2.22	0.06	2.34	0.07
19	4	18.10	1.05	18.11	0.16	2.54	0.48	2.31	0.02	1.78	0.03	1.73	0.16	2.07	0.17	2.26	0.08
20	1	19.05	–	19.08	–	1.79	–	2.37	–	1.68	–	1.61	–	1.94	–	2.71	–
21	1	20.92	–	18.05	–	2.51	–	2.86	–	1.79	–	1.75	–	2.23	–	2.42	–
22	2	23.32	0.01	23.01	0.01	2.41	0.01	2.93	0.01	2.13	0.04	1.77	0.01	2.78	0.01	2.95	0.07
23	2	20.52	0.71	21.09	2.39	3.46	0.31	3.62	0.19	2.31	0.08	2.13	0.15	2.41	0.22	2.91	0.16
24	4	22.85	2.00	22.07	2.51	3.35	0.39	3.27	0.22	2.46	0.27	2.15	0.31	2.72	0.23	2.68	0.17
25	1	21.51	–	22.50	–	2.80	–	3.54	–	2.17	–	2.25	–	2.26	–	2.94	–
26	1	28.03	–	28.89	–	3.57	–	3.45	–	2.26	–	2.41	–	3.16	–	3.00	–
27	0	–	–	–	–	–	–	–	–	–	–	–	–	–	–	–	–
28	2	29.52	0.08	30.82	0.11	4.37	0.06	4.80	0.13	2.69	0.01	2.55	0.12	3.65	0.13	3.35	0.10
29	1	27.18	–	27.42	–	4.28	–	4.60	–	2.94	–	2.55	–	2.86	–	3.01	–
30	0	–	–	–	–	–	–	–	–	–	–	–	–	–	–	–	–

Since the fetuses did not represent adequate samples for statistical analysis between particular 1-week intervals, we tested sex and laterality differences for the whole sample, without considering fetal ages

**Table 4:** Projection surface area and volume of the fetal clavicle in males

Gestational age (weeks)	*N*	Morphometric parameters of the male clavicle							
		Projection surface area (mm^2^)				Volume (mm^3^)			
		Right		Left		Right		Left	
		Mean	SD	Mean	SD	Mean	SD	Mean	SD
18	1	27.80	–	32.39	–	61.44	–	73.20	–
19	2	22.90	2.40	24.50	1.41	53.57	5.30	56.68	2.06
20	2	26.75	0.64	27.11	3.97	62.47	2.05	64.30	11.89
21	2	40.90	0.00	40.70	0.42	101.02	0.58	96.24	2.74
22	0	–	–	–	–	–	–	–	–
23	1	44.60	–	43.20	–	108.38	–	104.98	–
24	5	48.56	6.43	48.30	6.46	120.01	13.85	120.48	18.19
25	0	–	–	–	–	–	–	–	–
26	1	65.00	–	63.60	–	162.50	–	163.45	–
27	4	65.78	9.16	66.20	5.63	165.76	24.85	180.19	20.56
28	0	–	–	–	–	–	–	–	–
29	0	–	–	–	–	–	–	–	–
30	3	66.93	9.96	68.47	6.21	183.87	34.96	191.71	17.40

Since the fetuses did not represent adequate samples for statistical analysis between particular 1-week intervals, we tested sex and laterality differences for the whole sample, without considering fetal ages

**Table 5:** Projection surface area and volume of the fetal clavicle in females

Gestational age (weeks)	*N*	Morphometric parameters of the female clavicle							
		Projection surface area (mm^2^)				Volume (mm^3^)			
		Right		Left		Right		Left	
		Mean	SD	Mean	SD	Mean	SD	Mean	SD
18	2	32.81	1.40	33.91	0.72	74.65	3.42	77.49	1.89
19	4	24.45	0.30	25.01	1.01	55.39	1.39	56.61	2.98
20	1	27.10	–	29.90	–	63.14	–	72.06	–
21	1	33.50	–	29.90	–	81.07	–	70.03	–
22	2	45.30	0.14	39.80	0.14	111.89	0.99	94.73	0.90
23	2	41.75	8.56	41.25	7.42	101.21	20.45	99.54	17.06
24	4	47.65	10.05	46.15	8.08	125.82	27.51	121.17	20.55
25	1	40.70	–	40.10	–	102.56	–	98.25	–
26	1	71.20	–	65.40	–	180.14	–	164.81	–
27	0	–	–	–	–	–	–	–	–
28	2	75.70	3.54	71.90	3.68	199.41	6.64	183.40	11.41
29	1	65.50	–	54.60	–	168.34	–	151.79	–
30	0	–	–	–	–	–	–	–	–

Since the fetuses did not represent adequate samples for statistical analysis between particular 1-week intervals, we tested sex and laterality differences for the whole sample, without considering fetal ages

In the studied age range, the mean projection surface area of the clavicle increased from 31.14 ± 3.06 to 66.93 ± 9.96 mm^2^ on the right and from 33.40 ± 0.02 to 68.47 ± 6.21 mm^2^ on the left, following the function *y* = −51.078 + 4.174 × ln(age) ± 6.943 (*R* ^2^ = 0.82)—(Fig. 4e), with fetal ages expressed in weeks.

On the right and left sides, the mean volume of the clavicle increased from 70.24 ± 8.00 to 183.87 ± 34.96 mm^3^ and from 76.06 ± 2.81 to 191.71 ± 17.40 mm^3^, respectively, following the function *y* = −766.948 + 281.774 × ln(age) ± 19.610 (*R* ^2^ = 0.84)—(Fig. 4f), where age was expressed in weeks.

## Discussion

As reported in the professional literature, to date, assessment of the development of the clavicle has involved different research methods: anatomical dissection, anthropometric measurements, X-rays, ultrasound, and 3D-ultrasound, but a CT examination has not been used yet. Therefore, we decided to perform a precise quantitative assessment of the clavicle in human fetuses based on objective research techniques, i.e., computed tomography and digital image analysis. Of note, our findings have been presented as if describing a developmental sequence in one fetus, even though the numerical data have truly been cross-sectional, resulting from 42 autopsied fetuses.

The study revealed neither sex nor bilateral differences regarding the examined morphometric parameters of the clavicle. The lack of differences between the right and left fetal clavicles was also confirmed by Mohsin et al. [3]. On the contrary, both sex and bilateral differences were found in adults, since numerous authors [10–13] reported clavicle length to be greater in men than in women. Albeit preponderance of researchers [4, 8, 10, 11, 13–16] reported the left clavicle to be consequently longer than the right one, only Mays et al. [16] and Auerbach and Raxter [14] proved statistically significant right–left differences. We share the view of Mohsin et al. [3] that in the fetus, the right and left clavicles develop symmetrically. The subsequent bilateral differences observed in adults are related with the development of the dominant upper limb, type of work, and everyday life conditions. However, the literature lacks quantitative data on clavicle length in fetuses of different ethnic origin [2, 3, 9, 17].

In this study, based on autopsied human fetuses aged 18–30 weeks, the mean lengths of the right and left clavicles increased from 16.97 to 28.70 mm, and from 18.94 to 29.39 mm, respectively. According to Mohsin et al. [3], in their autopsy material consisting of 15 human fetuses aged 14–33 weeks, the mean lengths of the right and left clavicles increased from 14.12 to 34.58, and from 14.43 to 34.71 mm, correspondingly. Black and Scheuer [9] evaluated clavicle length in *in utero* fetuses using ultrasound, and on autopsy specimens using anthropometric techniques. The mean clavicle length in the former group increased from 17 mm at 16 weeks to 41 mm at 40 weeks, while in the latter group increased from 8.2 mm at 12 weeks to 44.1 mm at 40 weeks. In the material under examination, an increase in clavicle length has followed the function *y* = −54.439 + 24.673 × ln(age) that clearly corresponds with the findings by Sherer et al. [2]. These authors performed an ultrasound cross-sectional study of 623 consecutive patients between 14 and 42 weeks’ gestation, and proved an increase in clavicle length in accordance with the function *y* = −75.30 + 32.70 × ln(age) ± (–0.41 + 0.08328 × age). Nevertheless, 20 years earlier, Yarkoni et al. [17] had ultrasonically evaluated clavicle length in 85 human fetuses aged 15–40 weeks and reported a commensurate increase in its length to follow the function: *y* = 1.118303 + 0.9788639 × age. As it turned down, the clavicle length expressed in millimeters was roughly equivalent to gestational age in weeks—the so-called “simple 1 mm = 1-week rule”. Thus, as stated by these authors, clavicle length could be a useful parameter for the estimation of gestational ages and in the detection of congenital anomalies of the clavicle [17]. According to Sherer et al. [2], their own measurements were consistently substantially larger than those achieved by Yarkoni et al. [17], and so the latter could considerably overestimate gestational ages by as much as 6 weeks.

Our measurements unveiled that the mean width of the clavicle acromial end increased from 2.34 to 4.20 mm on the right, and from 2.46 to 4.48 mm on the left. As stated by Mohsin et al. [3], the width of the acromial end of the right and left clavicles increased from 1.92 ± 0.34 to 4.47 ± 0.53 mm, and from 2.00 ± 0.38 to 4.55 ± 0.64 mm, respectively. In the material under examination, the mean width of the clavicle central part increased from 1.77 to 2.69 mm on the right, and from 1.75 to 2.66 mm on the left. According to Mohsin et al. [3], this parameter increased from 1.08 ± 0.27 to 2.65 ± 0.28 mm on the right, and from 1.09 ± 0.22 to 2.67 ± 0.37 mm on the left. We found the width of the sternal end of the right and left clavicles to increase from 2.23 ± 0.04 to 3.23 ± 0.29 mm, and from 2.32 ± 0.06 to 3.09 ± 0.17 mm, correspondingly. As reported by Mohsin et al. [3], the widths of the right and left clavicle sternal ends increased from 1.43 ± 0.37 to 3.98 ± 0.59 mm, and from 1.45 ± 0.21 to 3.83 ± 0.72 mm, respectively. Of note, in this study, the three widths of the clavicle increased logarithmically.

Interestingly enough, in contrast to our study, Mohsin et al. [3] also focused on the vertical dimensions of the central part and both ends of the clavicle. The vertical dimensions of the right and left clavicles increased from 0.97 ± 0.23 to 2.08 ± 0.416 mm, and from 1.03 ± 0.30 to 2.06 ± 0.419 mm, respectively, for the acromial end; from 1.18 ± 0.104 to 2.07 ± 0.29 mm and from 1.13 ± 0.098 mm to 2.02 ± 0.30 mm, respectively, for the central part; as well as from 1.37 ± 0.15 to 4.58 ± 1.22 mm and from 1.54 ± 0.30 to 4.74 ± 1.03 mm, respectively, for the sternal end.

This study has been the first in the medical literature to quantitatively analyze both clavicle projection surface area and volume at varying gestational ages. In the fetuses of 18–30 weeks, the mean projection surface area of the right and left clavicles increased from 31.14 to 66.93 mm^2^ and from 33.40 to 68.47 mm^2^, respectively, which clearly corresponded to a commensurate growth, following the function *y* = −51.078 + 4.174 × age. Three-dimensional reconstructions of various organs and their volumetric analysis by adding together CT scans [6, 18–20] have encouraged us to examine the volume of the clavicle. Between 18 and 30 weeks of gestation, the mean clavicle volume increased from 70.24 to 183.87 mm^3^ on the right and from 76.06 to 191.71 mm^3^ on the left, following the function *y* = −766.948 + 281.774 × ln(age) ± 19.610.

The numerical data describing the clavicle growth in the human fetus obtained in this study may be of potential relevance. Obviously, the width of the shoulder girdle is determined by the clavicle length [17]. Furthermore, fetal macrosomia causes the width of the shoulder girdle to be greater than the dimensions of the head, impeding or even preventing a natural delivery [1]. Nevertheless, according to Sherer et al. [2], there were no systematic differences between clavicle length from diabetic (*n* = 22) and non-diabetic (*n* = 601) subjects. As reported by Chez et al. [21] and Lam et al. [22], clavicle fracture is considered an unavoidable event, accompanying fetal macrosomia, and shoulder dystocia. Sherer et al. [2] stated, however, that in spite of the apparent association between birth weight and clavicle fracture, no data are currently available referring to clavicle length and either shoulder dystocia or clavicle fracture. Perusing the clavicular length may help to predict the risks of the delivery and predisposition to clavicle fractures [17]. This is particularly germane, since the incidence of such fractures reaches 0.2–3.5%, constituting the second most common perinatal trauma after damages to head’s soft tissues [23]. Sherer et al. [2] do not suggest measuring clavicle length in all fetuses, but the overall presence and configuration of this important anterior component of the shoulder girdle should be noted, if possible.

To our opinion, ultrasound morphometric evaluation of the clavicle may be helpful in diagnosing many congenital defects, such as clavicular hypoplasia, often observed in cleidocranial dysplasia, incomplete ossification of the clavicle typical of Edwards syndrome (trisomy 18), and shortening of the clavicle observed in Holt–Oram syndrome, also known as Harris–Osborne syndrome, as well as in Goltz syndrome [1]. Melnick–Needles syndrome is characterized, among other things, by agenesis or bifurcation of the distal parts and curvatures of long bones, which referring to the clavicles results in narrow shoulders [1]. To date, computed tomography has not been used for the assessment of the clavicle development in human fetuses. Therefore, the presented results provide detailed knowledge concerning the growth models for the fetal clavicle. This may be of great importance in the prenatal diagnostics of congenital defects, malformations of the clavicle, and forensic medicine.

## Conclusions

1. In terms of morphometric parameters, the fetal clavicle shows neither sex nor bilateral differences.
2. The fetal clavicle increases logarithmically with respect to its length, width, and volume, and linearly with respect to its projection surface area.
3. The obtained morphometric data of the growing clavicle are considered normative for their respective weeks of gestation and may be of relevance in the diagnosis of congenital defects.
